# Design of Sodium Alginate/Gelatin-Based Emulsion Film Fused with Polylactide Microparticles Charged with Plant Extract

**DOI:** 10.3390/ma14040745

**Published:** 2021-02-05

**Authors:** Weronika Prus-Walendziak, Justyna Kozlowska

**Affiliations:** Faculty of Chemistry, Nicolaus Copernicus University in Torun, Gagarina 7, 87-100 Torun, Poland; weronika.pw@doktorant.umk.pl

**Keywords:** emulsion films, mechanical properties, moisture content, contact angle, skin assays, microparticles, encapsulation, *Calendula officinalis* flower extract

## Abstract

This study aimed at designing emulsion films based on sodium alginate, gelatin, and glycerol, and their modification by the addition of lipids (cottonseed oil and beeswax). Film composition with the most promising properties was further modified by the incorporation of polylactide (PLA) microparticles with *Calendula officinalis* flower extract. PLA microspheres were obtained by the emulsion/solvent evaporation method. The size distribution of oily particles in emulsions was investigated. Mechanical properties, moisture content, UV-Vis spectra, and the color of films were analyzed, while biophysical skin parameters were assessed after their application to the skin. Moreover, the contact angles were measured, and the surface free energy of polymeric films was determined. An investigation of the amount of *Calendula officinalis* flower extract which can be incorporated into PLA microparticles was performed. The modification of the composition of films significantly influenced their physicochemical properties. The selected active ingredient in the form of plant extract was successfully incorporated into polymeric microparticles that were further added into the developed emulsion film. The condition of the skin after the application of obtained emulsion films improved. The prepared materials, especially containing microparticles with plant extract, can be considered for designing new cosmetic forms, such as cosmetic masks, as well as new topical formulations for pharmaceutical delivery.

## 1. Introduction

**Figure 1:**
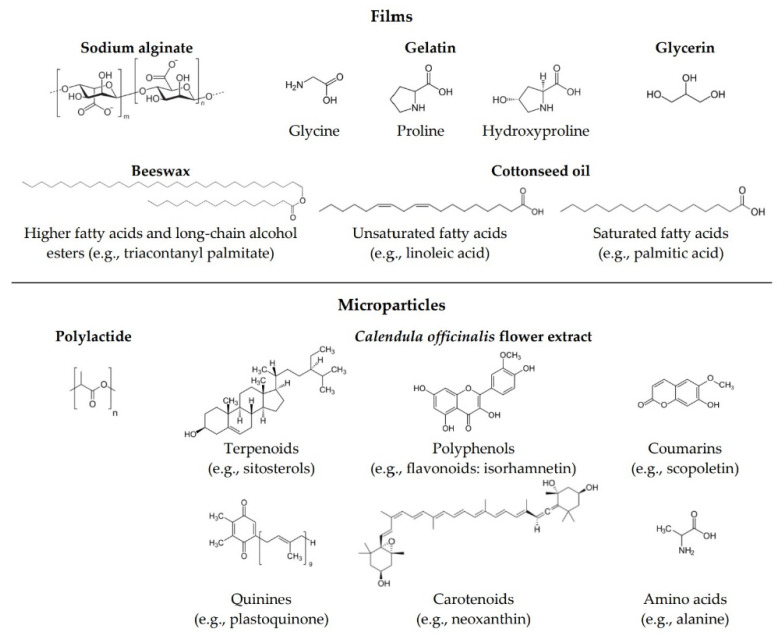
The structure of sodium alginate, glycerin and main constituents of gelatin, beeswax and cottonseed oil used for preparation of films; polylactide for encapsulation; main constituents of *Calendula officinalis* flower extract enclosed in microparticles.

Throughout the years, various alternatives for replacing non-biodegradable polymers have been investigated in order to face the increasing problem associated with environmental pollution and the use of plastic. Today’s materials should meet several requirements, such as biodegradability, non-toxicity, and biocompatibility. Hence, thin films made from biopolymers have recently attracted more attention [1,2,3,4]. Natural hydrophilic polysaccharides and proteins such as sodium alginate and gelatin (Figure 1), respectively, are widely used in cosmetics and materials science due to their thickening, gelling, and film-forming properties [5,6,7]. Alginates are linear, block copolymers composed of α-L-guluronic and β-D-mannuronic acid residues connected by a glycosidic bond [8]. Due to their structure, alginates belong to the group of anionic polysaccharides. Alginates are obtained from the cell walls of marine algae, mainly brown algae (*Phaeophyceae*) [8,9]. Gelatin is a denatured, biodegradable, high molecular weight polypeptide derived by processing of collagen extracted from animal tissue such as skin, muscle, and bone. The gelatin used commercially is predominantly obtained from by-products of the meat and leather industries, mainly bovine or porcine skin and bones, which are subjected to partial acid hydrolysis (giving gelatin type A) or partial alkaline hydrolysis (leading to gelatin type B) [10]. Gelatin contains all the essential amino acids except for tryptophan.

Properties of biopolymer films may be enhanced by adding an oily phase [11,12,13]. Emulsions are dispersed systems composed of two mutually immiscible liquids—the internal (the dispersed phase) is finely dispersed in the external (continuous) phase with the help of emulsifiers, which lowers the surface tension between two phases. Emulsions are widely used in the cosmetic, pharmaceutical, medical, and food industries [14,15,16]. Emulsions generally exhibit certain advantages mainly in that they enhance the therapeutic effect on the skin. Thin emulsion films prepared by the solution casting method have recently garnered more and more attention in the packaging and food industries [17,18,19,20].

Cottonseed oil is extracted from the cotton seeds through mechanical processes, such as crushing or pressing, or by chemical processes such as solvent extraction. Cottonseed oil is composed of 70% unsaturated fatty acids, including 18% monounsaturated, mainly oleic acid, and 52% polyunsaturated, predominantly linoleic acid. It also comprises 26% saturated fatty acids, mostly palmitic acid [21]. The relatively high level of saturated fatty acids contributes to the oxidative stability of cottonseed oil by offsetting the instability of the unsaturated fatty acid components [22,23]. In addition to skin conditioning properties, cottonseed oil also acts as an antioxidant due to tocopherol content [24]. Another commonly used oily substance is beeswax, a natural wax formed by honeybees of the genus *Apis*. Beeswax has a complex composition—it comprises over 300 different substances. Beeswax predominantly comprises 67% higher fatty acids and various long-chain alcohol esters, 14% hydrocarbons, and 12% free acids [25].

Microparticles are particles with diameters varying from 1 to 1000 μm in which the active substance is incorporated into the matrix. Many different materials can be used as encapsulating matrices, such as proteins, polysaccharides, fats, waxes, biopolymers, co-polymers or synthetic polymers [26]. One of the most promising polymers for encapsulation is polylactide (PLA) (Figure 1). It is a biocompatible and biodegradable polyester that is derived from renewable resources [27]. Polymeric encapsulation techniques include emulsion/solvent evaporation [28], extrusion [29], spray-drying [30], coacervation [31], and layer-by-layer [32,33]. Encapsulation technologies have a wide range of applications in many industrial sectors due to their undoubted advantages. Isolation and protection from external factors and undesired reactions (e.g., oxidation or deactivation) of active substances incorporated into microparticles are the main advantages of encapsulation. This also contributes to the maintenance of their stability. Encapsulation is used to enclose various types of solid and liquid materials such as drugs, extracts, vitamins, perfumes, proteins, dyes, bacterial cells, and essential oils, etc. [34,35,36,37,38,39]. *Calendula officinalis* flower extract, owing to its complex composition (Figure 1), possess multiple activities, including antioxidant, antibacterial, antifungal, antiviral, anti-inflammatory, antioedematous, and wound healing activities, as well as immunostimulant, anticancer, hepatoprotective, and insecticidal properties [40,41,42,43,44,45]. For these reasons, pot marigold is used in the treatment of numerous skin injures, such as burns, ulcers, skin inflammations, eczema, bruises, cuts, and abrasions of the epidermis, rashes, skin wounds, frostbites, and other conditions [46,47,48,49,50,51]. It is also an ingredient used for cosmetic purposes for dry, flaky, cracked, and prone to infection or redness skin [52,53,54]. *Calendula officinalis* is therefore a versatile and valuable herbal medicine used both externally and internally, which results from its rich and diverse chemical composition.

To the best of our knowledge, microparticles containing polyphenolic compounds, such as *Calendula officinalis* flower extract, have not yet been incorporated into emulsion films intended for cosmetic applications, as well as the effect of this type of materials on biophysical skin parameters remains unexplored.

The main goal of the current work was to design, prepare, and characterize thin emulsion films, as well as optimize their composition. Materials based on sodium alginate, gelatin, glycerol, and lipids (cottonseed oil and beeswax) were obtained by the solution casting method. The material composition with the most promising properties was subjected to further modification—the addition of PLA microparticles with *Calendula officinalis* flower extract—in order to create a material that has the ability to protect enclosed plant extract and that most effectively improves the condition of the skin. We analyzed the size distribution of oily particles in emulsion. The obtained films were characterized by moisture content, contact angle, mechanical properties, UV-Vis, and colorimetry. Biophysical skin parameters after the application of films to the skin were measured.

## 2. Materials and Methods

### 2.1. Materials

Polylactide was obtained from NatureWorks (Minnetonka, MN, USA). Gelatin from porcine skin (type A), Span 80, poly(vinyl alcohol) (product no 363138; the molecular weight was 31,000–50,000 g/mol and the hydrolysis degree was 98–99%), beeswax, Folin–Ciocalteu reagent, gallic acid and diiodomethane were acquired from Sigma-Aldrich (Poznan, Poland). Sodium alginate was purchased from BÜCHI Labortechnik AG (Flawil, Switzerland). Dichloromethane and glycerol were supplied from Avantor Performance Materials Poland S.A. (Gliwice, Poland). Sodium carbonate and ethyl alcohol were purchased from Stanlab (Lublin, Poland). Sodium hydroxide was acquired from Chempur (Piekary Slaskie, Poland). Cottonseed oil was provided by Alston Nova, S.L. (Barcelona, Spain). All used chemicals were of analytical grade. The 2% hydroglycolic *Calendula officinalis* flower extract (propylene glycol/water (80:20)) was obtained from Provital S.A. (Barcelona, Spain) and its total polyphenol content was assessed with the use of the Folin–Ciocalteu method. The absorbance was measured at 725 nm using a UV–VIS spectrophotometer and the results were calculated as gallic acid equivalents (mgGAE/mL). The total polyphenolic content of the extract was 206.6 ± 3.6 mgGAE/mL.

### 2.2. Production of Polylactide Microparticles

Polylactide (PLA) microparticles loaded with *Calendula officinalis* flower extract were fabricated using the emulsion/solvent evaporation technique. Firstly, PLA was dissolved in dichloromethane (DCM) at a concentration of 5% (*w*/*v*). Meanwhile, a 1% (*w*/*v*) of poly(vinyl alcohol) (PVA) solution in 0.1% hydroglycolic *Calendula officinalis* flower extract was prepared. The organic phase containing PLA was slowly added to the PVA solution, with a volume ratio of 1:10 (PLA:PVA), during five minutes under magnetic stirring at 800 rpm. Subsequently, it was stirred continuously for four hours at room temperature to allow the DCM evaporation. The *Calendula officinalis* flower extract-loaded PLA microparticles were collected by centrifugation at 10,000 rpm for 10 min. After centrifugation, the precipitated microspheres were washed with deionized water to remove the residual PVA. The centrifugation and rinsing processes were repeated three times.

### 2.3. Production of Emulsion Films

Emulsion films were fabricated by using the solution casting method. Firstly, two solutions were made: an aqueous phase containing sodium alginate (ALG), gelatin (GEL), and different amounts of glycerol (G), and an oily phase consisting of cottonseed oil, beeswax, and emulsifier Span-80. Both phases were heated (70–80 °C), and subsequently mixed and homogenized (20,000 rpm, 3 min) (T25 digital ULTRA-TURRAX disperser, IKA Werke, Staufen, Germany). The film-forming solutions were cast on glass plates and dried (25 °C, 72 h). Moreover, the control samples, which did not contain the oily phase, were also prepared: ALG/GEL/G (1%) and ALG/GEL/G (10%).

After all the samples were characterized, one sample with the highest potential in terms of cosmetic chemistry was selected, to which PLA microparticles were added. In order to obtain microparticles-loaded emulsion film, the PLA microspheres containing *Calendula officinalis* flower extract were added to the film-forming solution before casting.

**Figure 2:**
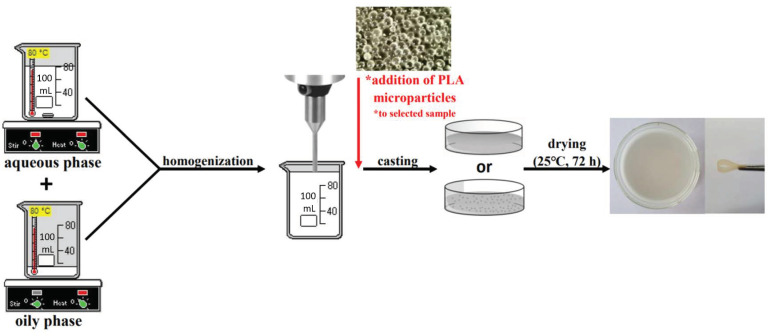
The scheme of production of emulsion films.

The composition of prepared materials is presented in Table 1, while the scheme of preparing materials is presented in Figure 2.

### 2.4. Characterization of Materials

#### 2.4.1. Depiction of PLA Microparticles and Materials

The microspheres’ morphology was observed by the optical microscope Delta Optical L-1000 (Minsk Mazowiecki, Poland).

#### 2.4.2. Emulsion Particles Size Distribution

The particle size distribution of emulsions based on sodium alginate, gelatin, glycerin, and lipids (cottonseed oil and beeswax) was measured using a Laser Diffraction Particle Size Analyzer (SALD-2300 with sampler SALD-MS23, Shimadzu, Kyoto, Japan). Small volumes of emulsions were added to the distilled water in the dispersion bath in the sampler. The dispersed particles were measured as they were circulated between the flow cell and the dispersion bath. The particles in the measurement unit were irradiated with a laser beam, and the particle size distribution was calculated using the light intensity distribution of scattered light generated from sample particles.

#### 2.4.3. Loading Capacity of Materials with PLA Microparticles

The Folin–Ciocalteu test was employed to determine the loading capacity of *Calendula officinalis* flower extract contained in the emulsion film with PLA microparticles [55]. The weighed 1 × 1 cm specimens of the film were put into 2 mL of 1 M NaOH for 1 h and, subsequently, they were centrifuged (10,000 rpm, 5 min). Afterwards, 20 µL of the supernatant solutions were mixed with 1.58 mL distilled water, 100 µL Folin–Ciocalteu reagent, and, after 4 min, 300 µL saturated Na_2_CO_3_ solution. The prepared mixtures were incubated at 37 °C for 40 min until a characteristic blue color was obtained. The absorbance was measured at 725 nm using a UV-Vis spectrophotometer (UV-1800, Shimadzu, Kyoto, Japan). The presented data of polyphenol content in the film with PLA microspheres were calculated using the calibration curve for the standard solution (gallic acid) in the concentration range of 0–0.50 mg/mL (R = 0.9995). The measurement was conducted in triplicate.

#### 2.4.4. Contact Angle and Surface Free Energy Measurements

The contact angle (θ) measurements were carried out with two liquids (diiodomethane and glycerol) using a DSA G10 goniometer equipped with a system of drop shape analysis (Krüss GmbH, Hamburg, Germany) at a constant temperature (25 °C). Seven measurements of each sample were performed. Due to the significant surface roughness of the film containing microparticles, only films without the addition of microspheres were subjected to contact angle measurements.

The surface free energy and its polar and dispersive components were calculated with the use of the Owens–Wendt method, which is based on the Young Equation (1). When a liquid comes into contact with a solid in a gaseous phase, there is a relationship between the contact angle θ, the surface tension of the liquid γ_lg_, the interfacial tension between the liquid and solid γ_sl_, and the surface free energy of the solid γ_sg_:

γ_sg_ = γ_sl_ + γ_lg_·cosθ (1)

#### 2.4.5. Film Color and Opacity

The color values (expressed as CIELAB color space: L* (0 black to 100 white), a* (−greenness to +redness) and b* (−blueness to +yellowness)) of the films were measured with the use of a colorimeter (Skin-Colorimeter CL 400, Courage + Khazaka, Köln, Germany). The measurements were performed in triplicate on the white background (L* = 88.49 ± 0.09, a* = −0.77 ± 0.04, b* = −0.74 ± 0.06). The total difference of color (ΔE*) (2) and whiteness index (WI) (3) of films were calculated using the following equations [56]:

(2) $$\Delta E=\;\sqrt{{\left(\Delta L^{*}\right)}^{2}+{\left(\Delta a^{*}\right)}^{2}+{\left(\Delta b^{*}\right)}^{2}}$$

(3) $$\mathrm{WI}=\;100-\sqrt{{\left(100-L^{*}\right)}^{2}+{\left(a^{*}\right)}^{2}+{\left(b^{*}\right)}^{2}}$$

where ΔL*, Δa* and Δb* are the differences between the color value of the samples and the results of the white background.

The light barrier properties of the films were studied at wavelengths ranging between 200 and 800 nm using a UV-Vis spectrophotometer (UV-1800, Shimadzu, Kyoto, Japan). The opacity (Op) (4) of the films was calculated as the absorbance at 600 nm (A_600_) divided by the film’s thickness (x; mm) [57]:

Op = A_600_/x (4)

#### 2.4.6. Moisture Content Measurements

Film samples (2 × 2 cm) were weighed before (W*_w_*) and after (W*_d_*) drying at 105 °C for 24 h [58]. The measurements were performed in triplicate. The moisture content (5) was calculated as the percentage of water loss from each sample as follows:

moisture content = (W*_w_* − W*_d_*)/W*_w_* × 100 (5)

#### 2.4.7. Mechanical Properties

A mechanical testing machine (Shimadzu EZ-Test EZ-SX, Kyoto, Japan) fitted with a 50 N load cell was used to test the mechanical properties of films. Dumbbell-shaped samples with known width and thickness were inserted into tensile grips and stretched until the break with the velocity of 5 mm/min. Young’s modulus was calculated from the slope of the stress-strain curve in the linear region (strain from 0.5% to 1.0%). The results were recorded using the Trapezium X Texture program. Average values were calculated using seven measurements for each sample.

#### 2.4.8. Biophysical Skin Parameters Assays

The skin color, skin surface hydration, and skin barrier quality (manifested as transepidermal water loss—TEWL) after application of the materials were examined using the colorimeter (Skin-Colorimeter CL 400, Courage + Khazaka, Köln, Germany), corneometer (Corneometer CM 825, Courage + Khazaka, Köln, Germany) and tewameter (Tewameter TM 300, Courage + Khazaka, Köln, Germany), respectively, with the use of MPA-software [59]. The skin parameter measurements were conducted on the volar forearm skin with the participation of five probands with normal skin (women, aged 24–27) in a controlled temperature (20–22 °C) and humidity (relative humidity 40–60%). All subjects gave their informed consent for inclusion before they participated in the study. The study was conducted in accordance with the Declaration of Helsinki, and the protocol was approved by the Ethics Committee of KB 67/2021.

Four 4 × 4 cm sections were used on the skin of both forearms. One section was the control field, and the remaining seven places were covered with samples of films previously immersed in water. The individual samples were applied at one-minute intervals, one after the other. The samples were removed from the skin 10 min after application.

The corneometric measurements were performed immediately after removal of the films that had been applied to the skin for 10 min followed by 15 min, 30 min, 1 h, 2 h, 3 h and 4 h after removing the samples as compared to the untreated control. The area of the untreated forearm served as a negative control. For each proband, five corneometric measurements were made on each of the eight skin areas at each time point. The results show the difference in the corneometer indications between the test field and control field results at the appropriate point.

Tewametric and colorimetric tests do not require simultaneous testing on the control and treated areas at each time point. TEWL and skin color analyses were examined with the initial measurements (control) before applying each film. Measurements on the seven tested areas were carried out in triplicate for each proband before applying films (seven control values), then immediately after removing the film from the skin and 15 min, 30 min, 1 h, 2 h, 3 h and 4 h after removing the samples.

#### 2.4.9. Statistical Analysis

One way ANOVA with Dunnett’s post hoc analysis was performed to statistically compare results. The results of moisture content and skin condition assays (hydration and TEWL) for emulsion films were compared to the control samples without lipids: ALG/GEL/G (1%) and ALG/GEL/G (10%). Meanwhile, the results of skin color were compared to the results made for the control field (before the start of the study) in order to assess significant changes in the skin redness before and after application of films to the skin. GraphPad Prism 8 (GraphPad Software, San Diego, CA, USA) was used for all analyses. Data are shown as the mean ± S.D. for each experiment. *p*-values < 0.05 were considered significant.

## 3. Results and Discussion

### 3.1. Appearance and Structure of Materials

**Figure 3:**
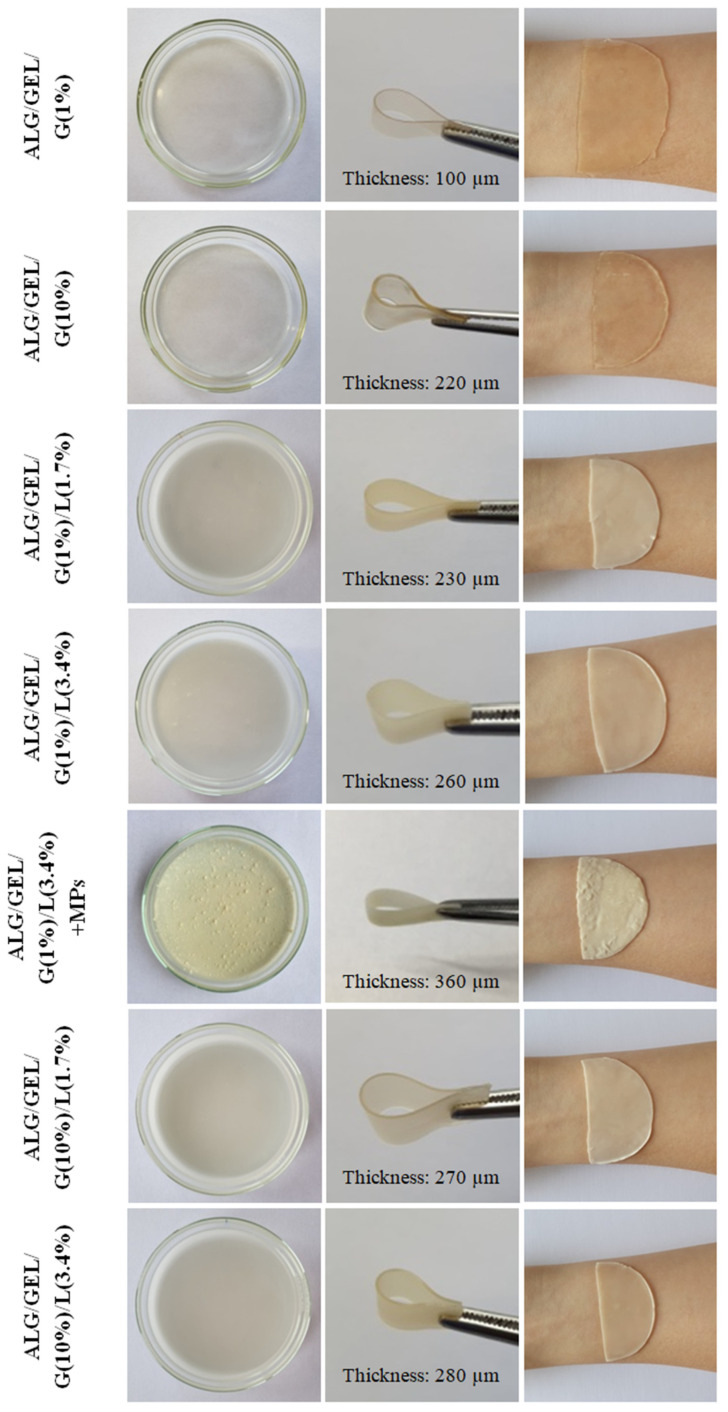
Photographs of prepared polymeric films and swollen polymeric films applied on the volar forearm skin.

Photographs of prepared polymeric films and swollen polymeric films applied on the volar forearm skin are presented in Figure 3. Films with a higher amount of glycerol, which acts as a plasticizer, were more flexible and sticky. The sample containing 1% glycerol and 3.4% lipids (ALG/GEL/G (1%)/L (3.4%)) was selected to further modification. The emulsion film containing PLA microparticles with incorporated *Calendula officinalis* flower extract was more brittle than the one without microspheres. PLA microspheres were arranged irregularly and formed agglomerates in some places of the emulsion film.

Control samples without lipids were thinner (polymeric film with 1% and 10% addition of glycerol were 100 and 220 µm thick, respectively). Meanwhile, a larger amount of glycerol and lipids increased their thickness (the thickness of emulsion films was from 230 µm to 280 µm). The thickest was the emulsion film fused with PLA microparticles (360 µm). The thickness was measured with a digital dial thickness gauge at a resolution of 0.001 mm (Sylvac, Switzerland).

**Figure 4:**
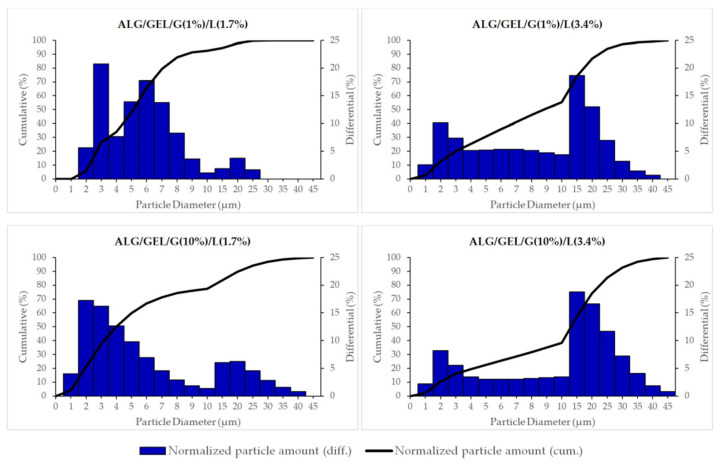
The size distribution of particles of prepared emulsion based on sodium alginate, gelatin, different amounts of glycerin, and lipids (cottonseed oil and beeswax).

The size distributions of oily particles in prepared emulsions based on sodium alginate, gelatin, different amount of glycerin and lipids (cottonseed oil and beeswax) are shown in Figure 4. Based on the obtained results, we can conclude that we obtained microemulsions. The results also indicate that the oily particles’ size distribution depends on the composition of emulsions, mainly on the amount of oily substances. The emulsions containing a lower amount of lipids had a lower mean size of particles (for ALG/GEL/G (1%)/L (1.7%), the mean diameter was 4.8 ± 0.2 µm and for ALG/GEL/G (10%)/L (1.7%), it was 4.5 ± 0.4 µm). Meanwhile, emulsions with a doubled amount of the oily phase had higher diameters of particles: ALG/GEL/G (1%)/L (3.4%) had 7.4 ± 0.4 µm particles and ALG/GEL/G (10%)/L (3.4%) had 9.9 ± 0.4 µm. The cumulative percentage of normalized particle amount reached a value of 50% at particle diameters of 5.1 and 4.0 µm for emulsions containing a smaller amount of lipids, such as ALG/GEL/G (1%)/L (1.7%) and ALG/GEL/G (10%)/L (1.7%), respectively, as well as 8.8 and 13.1 µm for emulsions with doubled amount of lipids, namely ALG/GEL/G (1%)/L (3.4%) and ALG/GEL/G (10%)/L (3.4%), respectively.

**Figure 5:**
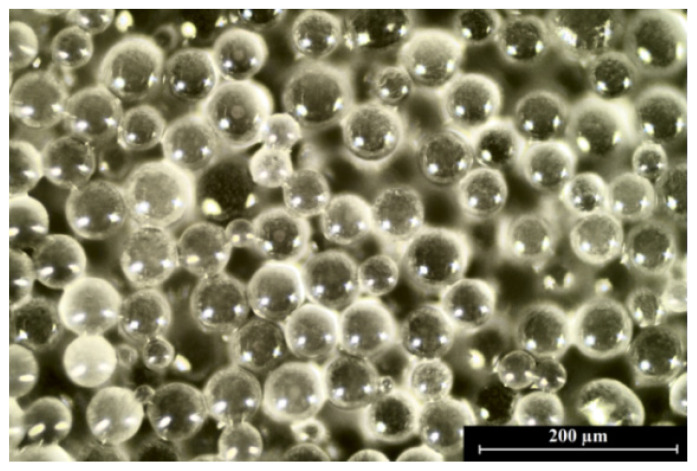
The image of PLA microparticles with *Calendula officinalis* flower extract observed by the optical microscope (scale bar = 200 µm).

The image of polylactide microspheres with *Calendula officinalis* flower extract observed by the optical microscope is presented in Figure 5. Prepared microparticles had a spherical shape and a very smooth surface. Their size was uniformly distributed (60.11 ± 10.94 µm).

The *Calendula officinalis* flower extract-loaded PLA microparticles were incorporated into emulsion films based on sodium alginate and gelatin with the addition of 1% of glycerol and 3.4% of lipids. The loading capacity of this material was examined by determining the content of polyphenolic compounds in the sample with the use of the Folin-Ciocalteu method. The *Calendula officinalis* flower extract was successfully incorporated into the emulsion film—the amount of polyphenols loaded into the emulsion film was 73 ± 7 mg/g.

### 3.2. Contact Angle and Surface Free Energy Measurements

The results of measurements of the contact angle, the surface free energy, and its dispersive and polar components calculated using the Owens–Wendt method for polymeric films are shown in Table 2. The emulsion film containing PLA microparticles was not subjected to this analysis due to its high surface roughness.

The contact angle technique is based on the measurement of non-covalent forces between the first monolayer of material and liquid [60]. The contact angles for diiodomethane (D) and glycerol (G) of obtained polymeric films were measured. A higher amount of glycerol led to an increase in contact angle values for both liquids in the case of polymer films without the addition of lipids. The addition of lipids in the film formulations caused a decline in the contact angle values for diiodomethane and glycerol. Moreover, in emulsion films, the decrease in the contact angles was also noticed for the samples with a higher glycerol amount.

The Owens–Wendt method was used for determining the surface free energy, as well as its polar and dispersive components. Based on the obtained results (Table 2), we noted that the sodium alginate/gelatin polymeric film with a 10% addition of glycerol (without lipids) possessed a hydrophobic surface as the polar component value was low for this sample. This may be attributed to the presence of interaction between gelatin and sodium alginate molecules which led to the existence of a lower number of polar groups on the blend surfaces. These interactions resulted from the established strong intermolecular hydrogen bonds formed within and between biopolymer chains involving their hydroxyl, carbonyl, and amino groups, as well as due to electrostatic interactions. It can be also concluded that the glycerol participated in the formation of intermolecular hydrogen bonds.

The measurements revealed that the polarity of the film surfaces significantly increased with the introduction of lipids into the film compositions. This indicates that the hydroxyl groups of the blend ingredients were directed towards the surface region and the formation of hydrogen bonds between the film components was disturbed in the presence of lipids. However, a doubled amount of lipids caused the opposite effect, leading to a decrease in the surface free energy and its polar component values, which is associated with the lower hydrophilicity of these samples’ surfaces. In the case of emulsion films, the higher amount of glycerol caused a rise in the values of surface free energy and its components.

### 3.3. Film Color and Opacity

**Figure 6:**
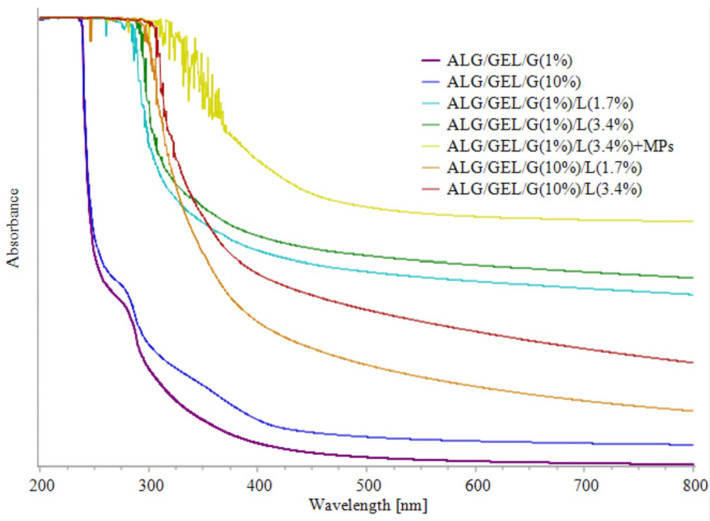
UV-Vis spectra of obtained films based on sodium alginate and gelatin with different amounts of glycerol and lipids, as well as the sample with incorporated PLA microparticles.

The UV-Vis spectra of fabricated films are shown in Figure 6. It was observed that the addition of lipids into sodium alginate/gelatin films can impact the light barrier properties. Control samples without lipids showed the lowest absorbance. The films exhibited a small absorbance peak at 275 nm that was extinguished in the spectra of emulsion films that contain lipids. The addition and increasing amount of lipids led to higher absorbance. The addition of PLA microparticles with *Calendula officinalis* flower extract increased the absorbance. Therefore, considering that the potential application of films could be as topical formulations for pharmaceutical and cosmetic delivery, the addition of microparticles provides higher UV light resistance.

On the basis of absorbance at 600 nm and film thickness, we calculated their opacity (Table 3). Higher opacity values represent lower transparencies of the films. It can be seen that emulsion films containing 10% glycerol were less opaque than the ones with a 1% addition of glycerol. The opacity of control samples (ALG/GEL/G (1%) and ALG/GEL/G (10%)) was significantly lower than that of emulsion films thus the addition of lipids increases this parameter.

Similarly, Aydogdu et al. prepared films from guar gum, glycerol, and the addition of orange oil for packaging applications [61]. They discovered that the opacity of their films ranged between ~4 and ~8 mm^−1^, and it increased significantly with orange oil incorporation and increased oil content from 1 to 2% *v/v*. They associated it with the increased light scattering by the dispersed oil droplets and, as a result, diffuse reflection increased which lowered film transparency.

The values of film color expressed as CIELAB color space (L*a*b*), as well as the calculated total difference of color value and whiteness of films are presented in Table 3. L* informs about the brightness of films, whereas a* about greenness/redness and b* about blueness/yellowness of tested films.

Changes in the film’s composition significantly altered the colorimeter indications. Emulsion films containing lipids were much more yellow compared to the control samples without the oily phase. The highest b* parameter responsible for the yellowness of tested films had a sample with PLA microparticles. Different amounts of lipids had no obvious impact on the film’s yellowness. In the case of a* values that locate the measuring values on the green-red axis, all the samples were shifted to slightly greener values. The higher a* indications had films containing 1% of glycerol and lipids. Based on the calculated total difference of color, one can see that the ΔE changed correspondingly to the opacity of films. Emulsion film with incorporated PLA microparticles had the highest value of total difference of color and the lowest value of whiteness index. However, the film’s whiteness index had the highest values for the control samples without lipids. The introduction of lipids caused a decrease in their whiteness.

Similar observations have been reported in other papers. Tongnuanchan et al. [17] and Xiao et al. [18] prepared palm oil-emulsified gelatin films. Tongnuanchan et al. found that with the increasing content of oil, the total difference of color and transparency of films increased, whereas the whiteness index decreased. They noted that the higher transparency value was in agreement with a lower L*-value and WI of films as oil level increased. Therefore, the incorporation of palm oil into their films directly influenced the transparency of films, whereas Xiao et al. observed the same dependencies but with an increasing degree of oil (degree is a commercial term referring to the melting point for palm oil). They attributed this to the higher crystallinity of oil and increased roughness of the film surface which interferes with light reflection.

### 3.4. Moisture Content Measurements

**Figure 7:**
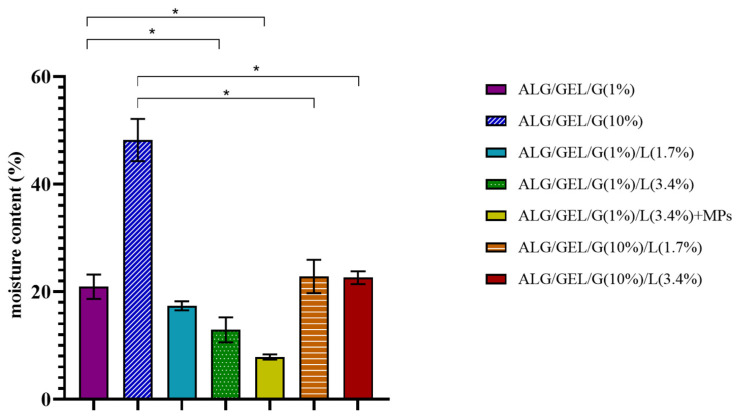
The moisture content of prepared polymeric films with different amounts of glycerol compared to the control samples (without lipids) (* indicates a difference at *p* < 0.05).

The results of moisture content after drying the samples at 105 °C for 24 h are presented in Figure 7. The measurements revealed that the moisture content of samples depends on the composition of emulsion films: the content of glycerol and lipids. The highest moisture content had polymeric film composed of sodium alginate, gelatin, and 10% glycerol (~48%). The higher amount of glycerol led to the higher moisture content in samples. This is due to the water-holding properties of glycerol so it could have attracted and retained a higher amount of water in the film matrix, primarily by hydrogen bonding. Our findings are in line with other studies investigating the moisture contained in polymeric films with different content of plasticizers [62,63].

The introduction of lipids into materials caused a drop in their moisture content. The doubled amount of lipids in samples containing 1% glycerol also led to a decrease in their moisture content; however, in samples with 10% addition of glycerol, we did not observe significant changes. Another important observation is that the addition of PLA microparticles into the film also decreased its moisture content, and thus ALG/GEL/G (1%)/L (3.4%) + MPs sample had the lowest moisture content (~8%).

### 3.5. Mechanical Properties

The values of Young’s modulus, maximum force, elongation at maximum force, break force, and elongation at break during tensile straining of polymeric films are shown in Table 4.

It can be noted that the samples containing a higher content of glycerol had considerably lower values of Young’s modulus and thus were significantly more flexible and broke later. This is due to the fact that glycerol acts as a plasticizer, and hence it reduces intermolecular hydrogen bonding while it increases intermolecular spacing [64,65].

The decrease in stiffness was also caused by the addition and an increase in the amount of added lipids, which resulted in a decrease in Young’s modulus values. This may be explained by the non-polymeric nature of lipids, which limits the cohesive film-forming capacity. Moreover, the presence of lipids in the emulsion films might have induced the development of heterogeneous structures causing discontinuities in the polymer network [65].

The incorporation of PLA microspheres into the emulsion film also led to the drop in Young’s modulus, and thus this sample was less stiff.

A corresponding impact of film composition was observed in the case of maximum force and break force. However, an opposite effect was noticed for elongation at maximum force and elongation at break. Elongation is a measure of the film’s capacity for stretching. The introduction of lipids in the formulations with 10% glycerol resulted in a decrease in stretchability. Meanwhile, the addition and doubling of lipids into the materials containing 1% of glycerol led to an increase in elongation at maximum force and at break. Cottonseed oil exhibited a plasticizing effect, which was revealed through the enhanced elongation of films containing 1% glycerol. This may be also due to the loss of cohesiveness and structural integrity of emulsion films [65].

Han et al. prepared emulsion films based on pea starch and glycerol with different amounts of beeswax [66]. They observed that the addition of beeswax affected the mechanical properties of films, significantly reducing tensile strength and elongation, as well as increasing elastic modulus. Limpisophon et al. also evaluated mechanical properties of the gelatin–fatty acid emulsion films, with varying fatty acid concentrations [67]. Their results revealed that the addition of fatty acid, from 0 to 100% of the protein concentration, significantly reduced the tensile strength of the film and increased their elongation at break.

### 3.6. Biophysical Skin Parameters Assays

**Figure 8:**
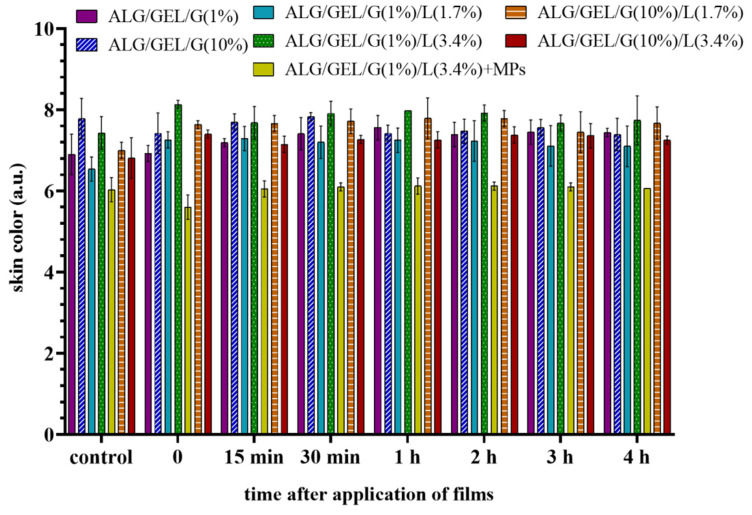
Skin color after application of films based on sodium alginate and gelatin with different amounts of glycerol and lipids, as well as the sample with incorporated PLA microparticles. The control measurements were performed before the application of materials to the skin, whereas time = 0 refers to the tests taken immediately after removal of the films that had been applied to the skin for 10 min. * indicates a difference at *p* < 0.05 between the results at an appropriate time compared to the results made for the control field.

**Figure 9:**
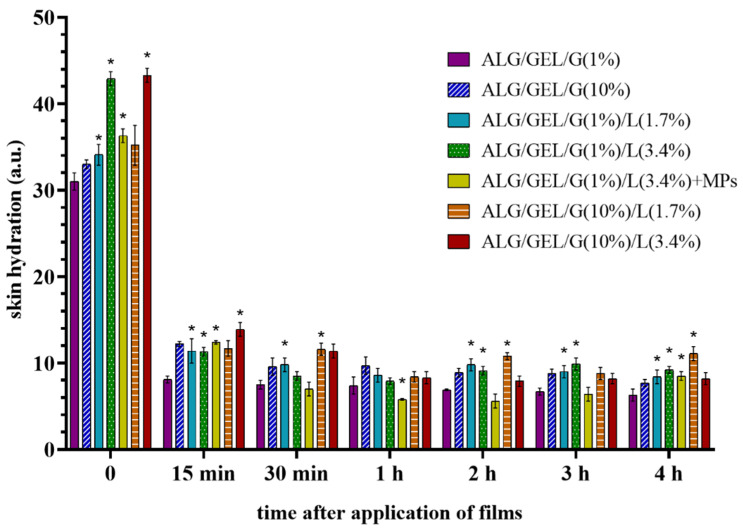
Skin hydration after application of films based on sodium alginate and gelatin with different amounts of glycerol and lipids, as well as the sample with incorporated PLA microparticles. Time = 0 refers to the measurements taken immediately after the removal of films from the skin. The results are the differences in the corneometer indications between the test field and control field at the appropriate point. Significant differences compared to the control samples: ALG/GEL/G (1%) and ALG/GEL/G (10%) for each time were marked on the graphs with (*).

**Figure 10:**
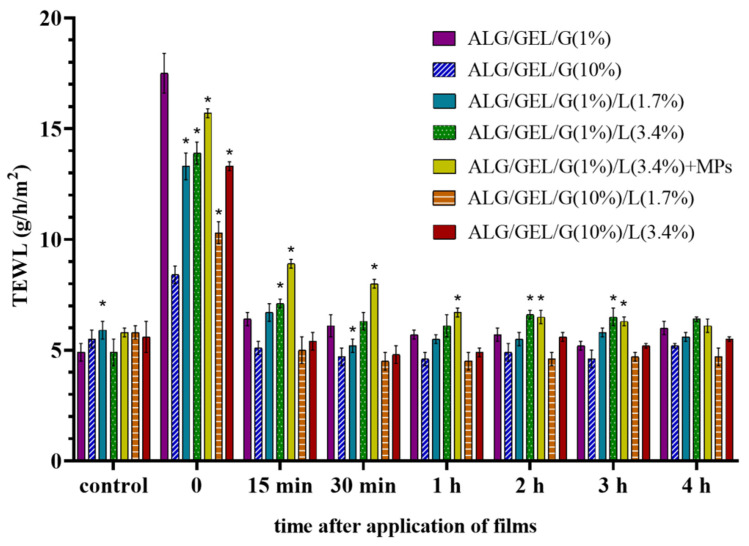
Skin barrier quality (transepidermal water loss—TEWL) after application of films based on sodium alginate and gelatin with different amounts of glycerol and lipids, as well as the sample with incorporated PLA microparticles. The control measurements were performed before the application of materials to the skin, whereas time = 0 refers to the tests taken immediately after removal of the films that had been applied to the skin for 10 min. Significant differences compared to the control samples: ALG/GEL/G (1%) and ALG/GEL/G (10%) for each time were marked on the graphs with (*).

The results of the analysis of the skin parameters after application to the skin of films are shown in Figure 8, Figure 9 and Figure 10. The biophysical skin parameters (including skin color, skin surface hydration and skin barrier quality) were examined with the use of Courage + Khazaka probes.

#### 3.6.1. Skin Color

The values of skin color are expressed as coordinates in the color space L*a*b*. L* informs about the skin brightness, a* locates the measured values on the red-green axis, whereas b* shows the color position on the blue-yellow axis and describes the skin pigmentation. a* values are proportional to skin redness, erythema, and microcirculation, and hence only they were considered in this investigation. The skin color was assessed by preliminary (control) measurements prior to the application of each film. The samples were applied to the skin for 10 min, and the colorimetric measurements were carried out at appropriate time intervals.

One can see that the application of obtained materials did not damage or irritate the skin, or cause a statistically significant change in skin redness (erythema) (Figure 8).

#### 3.6.2. Skin Hydration

Before the start of this analysis, the skin hydration level was measured in each of the eight designated fields on both forearms of five probands, and no significant differences between the corneometer indications were noticed in a given proband (data not shown). Skin surface hydration measurements were performed immediately after removal of the films that had been applied to the skin for 10 min and then 15 min, 30 min, 1 h, 2 h, 3 h and 4 h after removing the samples as compared to the untreated control. The results show the difference in the corneometer indications between the test field and control field results at each time point. The depth of corneometric measurement is very small and reaches solely to the stratum corneum in order to exclude the influence of deeper skin layers (e.g., from the blood vessels). The skin surface hydration indirectly determines its moisture [68].

As can be seen in Figure 9, the hydration level of the skin surface (stratum corneum) rocketed after the topical application of polymeric films. The lowest values of skin hydration caused the application of the control samples (without lipids). The addition and increase in the amount of lipids in films resulted in a rise in skin hydration levels. Therefore, the emulsion films containing a doubled amount of lipids led to the highest level of skin hydration (~43 a.u.). The introduction of microparticles into emulsion film slightly decreased its skin hydrating properties (the skin hydration immediately after its application was about 36 a.u.). The results indicate that films increase skin hydration in a process related to the occlusion effect [69]. However, 15 min after the application of samples, the skin hydration level went down. Until the end of the research, the corneometric indications were higher than the initial level measured before the application of films. Therefore, we can conclude that skin hydration after application of the prepared films compared to the skin hydration before the start of this analysis had statistically significantly improved. However, if we compare the moisturizing properties of emulsion films containing lipids to the ones without oily substances, we can notice that the introduction of lipids contributed to the level of skin hydration except for the sample containing 10% of glycerol and 3.4% of lipids (no significant differences were noticed 30 min after its removal from the skin). The incorporation of PLA microparticles with *Calendula officinalis* flower extract did not result in higher skin hydration properties compared to the sample with the same amount of glycerin and lipids (ALG/GEL/G (1%)/L (3.4%)).

#### 3.6.3. Transepidermal Water Loss (TEWL)

The permeability barrier function of the skin was assessed as transepidermal water loss (TEWL). The tewametric test started with the initial measurements (control) before applying each film followed by evaluation of TEWL immediately after removing the samples from the skin and 15 min, 30 min, 1 h, 2 h, 3 h and 4 h after removing the materials.

The results of the tewametric measurements were shaped in a similar manner as the skin hydration values (Figure 10). TEWL reached a high immediately after the topical application of prepared films owing to the temporary occlusion effect mentioned above. The highest TEWL was observed after the application of the control sample with 1% glycerol (17.5 g/h/m^2^). TEWL slumped after 15 min and began to stabilize as a result of the reorganization of the stratum corneum. Applying most films caused the TEWL values to approximately 5–6 g/h/m^2^, although the skin area for 3 h after removal of emulsion film fused with PLA microparticles had higher TEWL values.

The increase in TEWL results could have an indirect impact on corneometric measurements and it is caused by occlusion through films.

The application of prepared materials had a beneficial impact on biophysical skin parameters, which resulted from their composition. The skin structure loosens after the application of moist polymer materials due to the occlusion effect. Cottonseed oil and beeswax—emollients—present in films placed on the skin are occlusive moisturizers that prevent evaporative water loss to the environment. The stratum corneum moisture increases because water moving from the lower viable epidermal and dermal layers cannot penetrate through the stratum corneum. Additionally, polymers and glycerol act as moisturizing agents that are able to bind water. Polymers form an occlusive film, acting on the surface, which may result in higher TEWL. Reorganization of the stratum corneum resulted in higher skin hydration level, while no reddening of the skin and no permanent deterioration of the epidermal permeability barrier function were observed.

## 4. Conclusions

In this study, a microemulsion film fused with PLA microparticles charged with *Calendula officinalis* flower extract was successfully obtained. The results of this study demonstrate that the changes in the composition of films based on sodium alginate, gelatin, and glycerol significantly affected their properties. The introduction of lipids (cottonseed oil and beeswax) into material formulations led to a decrease in their moisture content, whereas it increased the hydrophilicity of their surfaces. Emulsion films with a greater addition of glycerol were more transparent and were characterized by greater flexibility, moisture content, thickness and higher values of surface free energy and its components. Test results indicate satisfactory cosmetic efficiency of the formulation containing 1% glycerol, 3.4% lipids, and 6% PLA microparticles of the encapsulated *Calendula officinalis* flower extract. This emulsion film had higher UV light resistance and increased skin hydration, but had no significant effect on skin redness. 

## Figures and Tables

**Table 1:** The types of prepared materials composed of sodium alginate (ALG), gelatin (GEL), glycerol (G), and different amount of lipids (L): cottonseed oil, beeswax, and emulsifier Span-80, as well as the sample containing polylactide microparticles (PLA MPs).

Sample	Composition of Materials (% *w/w*)						Addition (% *w/w*)
	Aqueous Phase			Oily Phase			PLA MPs
	ALG	GEL	G	Cottonseed Oil	Beeswax	Span-80	
ALG/GEL/G (1%)	2	1	1	-	-	-	-
ALG/GEL/G (10%)	2	1	10	-	-	-	-
ALG/GEL/G (1%)/L (1.7%)	2	1	1	1.2	0.5	0.35	-
ALG/GEL/G (1%)/L (3.4%)	2	1	1	2.4	1	0.7	-
ALG/GEL/G (1%)/L (3.4%) + MPs	2	1	1	2.4	1	0.7	6
ALG/GEL/G (10%)/L (1.7%)	2	1	10	1.2	0.5	0.35	-
ALG/GEL/G (10%)/L (3.4%)	2	1	10	2.4	1	0.7	-

**Table 2:** The contact angles (°) of diiodomethane (D) and glycerol (G), the surface free energy (γ_s_), dispersive (γ_s_^d^) and polar (γ_s_^p^) components for polymer films based on sodium alginate, gelatin, and glycerol with the addition of lipids (calculated by Owens–Wendt method).

Sample	Contact Angle (°)		Surface Free Energy (γ_s_) (mJ/m^2^)	Dispersive (γ_s_^d^) and Polar (γ_s_^p^) Components (mJ/m^2^)	
	D	G		γ_s_^d^	γ_s_^p^
ALG/GEL/G (1%)	54.2	61.5	37.56	23.92	13.64
ALG/GEL/G (10%)	69.3	99.9	23.12	22.75	0.39
ALG/GEL/G (1%)/L (1.7%)	38.2	39.0	51.92	28.69	23.24
ALG/GEL/G (1%)/L (3.4%)	48.8	51.8	43.58	25.19	18.40
ALG/GEL/G (10%)/L (1.7%)	34.6	22.5	59.21	28.28	30.93
ALG/GEL/G (10%)/L (3.4%)	39.5	45.2	48.81	29.08	19.72

**Table 3:** The color values (L*a*b*), a total difference of color value (ΔE), whiteness index (WI) and opacity (Op) of prepared films based on sodium alginate and gelatin with different amounts of glycerol and lipids, as well as the sample with incorporated PLA microparticles.

Sample	L*	a*	b*	ΔE	WI	Op (A_600_/mm)
ALG/GEL/G (1%)	89.49 ± 0.04	−1.19 ± 0.07	5.78 ± 0.06	6.61 ± 0.05	87.95 ± 0.05	1.10 ± 0.03
ALG/GEL/G (10%)	85.39 ± 0.07	−1.45 ± 0.08	8.75 ± 0.10	10.00 ± 0.12	82.91 ± 0.11	1.30 ± 0.05
ALG/GEL/G (1%)/L (1.7%)	82.33 ± 0.14	−0.37 ± 0.03	14.07 ± 0.11	16.04 ± 0.15	77.41 ± 0.16	7.35 ± 0.11
ALG/GEL/G (1%)/L (3.4%)	82.70 ± 0.09	−0.85 ± 0.04	13.79 ± 0.07	15.64 ± 0.03	77.86 ± 0.03	7.05 ± 0.09
ALG/GEL/G (1%)/L (3.4%) + MPs	80.84 ± 0.03	−0.46 ± 0.02	20.44 ± 0.04	22.52 ± 0.03	71.98 ± 0.02	6.26 ± 0.15
ALG/GEL/G (10%)/L (1.7%)	86.58 ± 0.06	−2.33 ± 0.04	12.19 ± 0.10	13.16 ± 0.09	81.73 ± 0.05	2.84 ± 0.04
ALG/GEL/G (10%)/L (3.4%)	84.66 ± 0.03	−2.68 ± 0.02	14.62 ± 0.14	15.94 ± 0.13	78.64 ± 0.09	4.46 ± 0.06

**Table 4:** Mechanical properties (Young’s modulus (E_mod_), maximum force (F_max_), elongation at maximum force, break force (F_break_) and elongation at break) of films based on sodium alginate and gelatin with different amounts of glycerol and lipids, as well as the sample with incorporated PLA microparticles.

Sample	E_mod_ (MPa)	F_max_ (N)	Elongation at F_max_ (mm)	F_break_ (N)	Elongation at Break (%)
ALG/GEL/G (1%)	1350.0 ± 91.4	20.4 ± 1.8	1.2 ± 0.2	20.3 ± 2.0	4.8 ± 0.8
ALG/GEL/G (10%)	2.2 ± 0.7	0.5 ± 0	37.9 ± 3.0	0.3 ± 0	173.0 ± 7.4
ALG/GEL/G (1%)/L (1.7%)	547.8 ± 19.8	19.2 ± 1.5	2.0 ± 0.2	19.1 ± 1.6	8.3 ± 0.7
ALG/GEL/G (1%)/L (3.4%)	266.4 ± 15.9	13.3 ± 0.9	2.4 ± 0.2	13.2 ± 1.0	9.9 ± 0.8
ALG/GEL/G (1%)/L (3.4%) + MPs	27.8 ± 4.6	1.7 ± 0.2	3.3 ± 0.7	1.6 ± 0.2	13.5 ± 2.8
ALG/GEL/G (10%)/L (1.7%)	0.8 ± 0.2	0.4 ± 0	30.2 ± 0.8	0.2 ± 0	135.6 ± 6.2
ALG/GEL/G (10%)/L (3.4%)	1.4 ± 0.1	0.3 ± 0	20.7 ± 3.2	0.2 ± 0	114.6 ± 4.6

## Data Availability

The data presented in this study are available on request from the corresponding author.

## References

[1] Dou L., Li B., Zhang K., Chu X., Hou H. (2018). Physical properties and antioxidant activity of gelatin-sodium alginate edible films with tea polyphenols. Int. J. Biol. Macromol..

[2] Łopusiewicz Ł., Drozlowska E., Trocer P., Kostek M., Śliwiński M., Henriques M.H.F., Bartkowiak A., Sobolewski P. (2020). Whey protein concentrate/isolate biofunctional films modified with melanin from watermelon (*Citrullus lanatus*) seeds. Materials.

[3] Piñeros-Hernandez D., Medina-Jaramillo C., López-Córdoba A., Goyanes S. (2017). Edible cassava starch films carrying rosemary antioxidant extracts for potential use as active food packaging. Food Hydrocoll..

[4] Bekhit M., Arab-Tehrany E., Kahn C.J.F., Cleymand F., Fleutot S., Desobry S., Sánchez-González L. (2018). Bioactive films containing alginate-pectin composite microbeads with lactococcus lactis subsp. Lactis: Physicochemical characterization and antilisterial activity. Int. J. Mol. Sci..

[5] Gordon P.W., Brooker A.D.M., Chew Y.M.J., Wilson D.I., York D.W. (2010). Studies into the swelling of gelatine films using a scanning fluid dynamic gauge. Food Bioprod. Process..

[6] Mackie W., Noy R., Sellen D.B. (1980). Solution properties of sodium alginate. Biopolymers.

[7] Fu S., Thacker A., Sperger D.M., Boni R.L., Buckner I.S., Velankar S., Munson E.J., Block L.H. (2011). Relevance of rheological properties of sodium alginate in solution to calcium alginate gel properties. AAPS Pharmscitech.

[8] Tønnesen H.H., Karlsen J. (2002). Alginate in drug delivery systems. Drug Dev. Ind. Pharm..

[9] Pereira L., Sousa A., Coelho H., Amado A.M., Ribeiro-Claro P.J.A. (2003). Use of FTIR, FT-Raman and 13C-NMR spectroscopy for identification of some seaweed phycocolloids. Biomol. Eng..

[10] Harris P. (1990). Food Gels.

[11] Siewert C.D., Haas H., Cornet V., Nogueira S.S., Nawroth T., Uebbing L., Ziller A., Al-Gousous J., Radulescu A., Schroer M.A. (2020). Hybrid Biopolymer and Lipid Nanoparticles with Improved Transfection Efficacy for mRNA. Cells.

[12] Lin W., Mashiah R., Seror J., Kadar A., Dolkart O., Pritsch T., Goldberg R., Klein J. (2019). Lipid-hyaluronan synergy strongly reduces intrasynovial tissue boundary friction. Acta Biomater..

[13] Petrin T.H.C., Fadel V., Martins D.B., Dias S.A., Cruz A., Sergio L.M., Arcisio-Miranda M., Castanho M.A.R.B., Dos Santos Cabrera M.P. (2019). Synthesis and Characterization of Peptide-Chitosan Conjugates (PepChis) with Lipid Bilayer Affinity and Antibacterial Activity. Biomacromolecules.

[14] Gilbert L., Picard C., Savary G., Grisel M. (2013). Rheological and textural characterization of cosmetic emulsions containing natural and synthetic polymers: Relationships between both data. Colloids Surf. A Physicochem. Eng. Asp..

[15] Muschiolik G. (2007). Multiple emulsions for food use. Curr. Opin. Colloid Interface Sci..

[16] Schijns V.E.J.C., Strioga M., Ascarateil S. (2014). Oil-based emulsion vaccine adjuvants. Curr. Protoc. Immunol..

[17] Tongnuanchan P., Benjakul S., Prodpran T., Nilsuwan K. (2015). Emulsion film based on fish skin gelatin and palm oil: Physical, structural and thermal properties. Food Hydrocoll..

[18] Xiao J., Wang W., Wang K., Liu Y., Liu A., Zhang S., Zhao Y. (2016). Impact of melting point of palm oil on mechanical and water barrier properties of gelatin-palm oil emulsion film. Food Hydrocoll..

[19] Galus S., Kadzińska J. (2015). Food applications of emulsion-based edible films and coatings. Trends Food Sci. Technol..

[20] Spotti M.L., Cecchini J.P., Spotti M.J., Carrara C.R. (2016). Brea Gum (from *Cercidium praecox*) as a structural support for emulsion-based edible films. LWT Food Sci. Technol..

[21] Jahaniaval F., Kakuda Y., Marcone M.F. (2000). Fatty acid and triacylglycerol compositions of seed oils of five Amaranthus accessions and their comparison to other oils. J. Am. Oil Chem. Soc..

[22] Pazzoti G., Souza C., Veronezi C., Luzia D., Jorge N. (2018). Evaluation of Oxidative Stability of Compound Oils under Accelerated Storage Conditions. Braz. Arch. Biol. Technol..

[23] Taghvaei M., Jafari S.M., Assadpoor E., Nowrouzieh S., Alishah O. (2014). Optimization of microwave-assisted extraction of cottonseed oil and evaluation of its oxidative stability and physicochemical properties. Food Chem..

[24] El-Mallah M.H., El-Shami S.M., Hassanien M.M.M., Abdel-Razek A.G. (2011). Effect of chemical refining steps on the minor and major components of cottonseed oil. Agric. Biol. J. N. Am..

[25] Tulloch A.P. (1980). Beeswax—Composition and Analysis. Bee World.

[26] Esfanjani A.F., Jafari S.M., Assadpoor E., Mohammadi A. (2015). Nano-encapsulation of saffron extract through double-layered multiple emulsions of pectin and whey protein concentrate. J. Food Eng..

[27] Oh J.K. (2011). Polylactide (PLA)-based amphiphilic block copolymers: Synthesis, self-assembly, and biomedical applications. Soft Matter.

[28] Kizilbey K. (2019). Optimization of Rutin-Loaded PLGA Nanoparticles Synthesized by Single-Emulsion Solvent Evaporation Method. ACS Omega.

[29] Bamidele O.P., Emmambux M.N. (2020). Encapsulation of bioactive compounds by “extrusion” technologies: A review. Crit. Rev. Food Sci. Nutr..

[30] Ballesteros L.F., Ramirez M.J., Orrego C.E., Teixeira J.A., Mussatto S.I. (2017). Encapsulation of antioxidant phenolic compounds extracted from spent coffee grounds by freeze-drying and spray-drying using different coating materials. Food Chem..

[31] Tavares L., Noreña C.P.Z. (2020). Encapsulation of Ginger Essential Oil Using Complex Coacervation Method: Coacervate Formation, Rheological Property, and Physicochemical Characterization. Food Bioprocess Technol..

[32] Pan H.M., Yu H., Guigas G., Fery A., Weiss M., Patzel V., Trau D. (2017). Engineering and Design of Polymeric Shells: Inwards Interweaving Polymers as Multilayer Nanofilm, Immobilization Matrix, or Chromatography Resins. ACS Appl. Mater. Interfaces.

[33] Boudou T., Crouzier T., Ren K., Blin G., Picart C. (2010). Multiple functionalities of polyelectrolyte multilayer films: New biomedical applications. Adv. Mater..

[34] Li L., Zhang W., Peng J., Xue B., Liu Z., Luo Z., Lu D., Zhao X. (2020). A novel shell material-highland barley starch for microencapsulation of cinnamon essential oil with different preparation methods. Materials.

[35] Hategekimana J., Masamba K.G., Ma J., Zhong F. (2015). Encapsulation of vitamin E: Effect of physicochemical properties of wall material on retention and stability. Carbohydr. Polym..

[36] Cortial A., Vocanson M., Loubry E., Briançon S. (2015). Hot homogenization process optimization for fragrance encapsulation in solid lipid nanoparticles. Flavour Fragr. J..

[37] Mohan A., Rajendran S.R.C.K., He Q.S., Bazinet L., Udenigwe C.C. (2015). Encapsulation of food protein hydrolysates and peptides: A review. RSC Adv..

[38] Lv Y., Pan Z., Song C., Chen Y., Qian X. (2019). Locust bean gum/gellan gum double-network hydrogels with superior self-healing and pH-driven shape-memory properties. Soft Matter.

[39] Pavli F., Argyri A.A., Skandamis P., Nychas G.J., Tassou C., Chorianopoulos N. (2019). Antimicrobial activity of oregano essential oil incorporated in sodium alginate edible films: Control of Listeria monocytogenes and spoilage in ham slices treated with high pressure processing. Materials.

[40] Khalid K., Teixeira da Silva J. (2012). Biology of Calendula officinalis Linn.: Focus on pharmacology, biological activities and agronomic practices. Med. Aromat. Plant Sci. Biotechnol..

[41] John R., Jan N. (2017). Calendula Officinalis-An Important Medicinal Plant with Potential Biological Properties. Proc. Indian Natl. Sci. Acad..

[42] Preethi K.C., Kuttan G., Kuttan R. (2006). Antioxidant potential of an extract of Calendula officinalis flowers in vitro and in vivo. Pharm. Biol..

[43] Ukiya M., Akihisa T., Yasukawa K., Tokuda H., Suzuki T., Kimura Y. (2006). Anti-inflammatory, anti-tumor-promoting, and cytotoxic activities of constituents of marigold (*Calendula officinalis*) flowers. J. Nat. Prod..

[44] Efstratiou E., Hussain A.I., Nigam P.S., Moore J.E., Ayub M.A., Rao J.R. (2012). Antimicrobial activity of Calendula officinalis petal extracts against fungi, as well as Gram-negative and Gram-positive clinical pathogens. Complement. Ther. Clin. Pract..

[45] Jiménez-Medina E., Garcia-Lora A., Paco L., Algarra I., Collado A., Garrido F. (2006). A new extract of the plant calendula officinalis produces a dual in vitro effect: Cytotoxic anti-tumor activity and lymphocyte activation. BMC Cancer.

[46] Fonseca Y.M., Catini C.D., Vicentini F.T.M.C., Nomizo A., Gerlach R.F., Fonseca M.J.V. (2010). Protective effect of Calendula officinalis extract against UVB-induced oxidative stress in skin: Evaluation of reduced glutathione levels and matrix metalloproteinase secretion. J. Ethnopharmacol..

[47] Chandran P.K., Kuttan R. (2008). Effect of Calendula officinalis flower extract on acute phase proteins, antioxidant defense mechanism and granuloma formation during thermal burns. J. Clin. Biochem. Nutr..

[48] Vargas E.A.T., Do Vale Baracho N.C., De Brito J., De Queiroz A.A.A. (2010). Hyperbranched polyglycerol electrospun nanofibers for wound dressing applications. Acta Biomater..

[49] Okuma C.H., Andrade T.A.M., Caetano G.F., Finci L.I., Maciel N.R., Topan J.F., Cefali L.C., Polizello A.C.M., Carlo T., Rogerio A.P. (2015). Development of lamellar gel phase emulsion containing marigold oil (*Calendula officinalis*) as a potential modern wound dressing. Eur. J. Pharm. Sci..

[50] Pedram Rad Z., Mokhtari J., Abbasi M. (2019). Calendula officinalis extract/PCL/Zein/Gum arabic nanofibrous bio-composite scaffolds via suspension, two-nozzle and multilayer electrospinning for skin tissue engineering. Int. J. Biol. Macromol..

[51] Aro A.A., Perez M.O., Vieira C.P., Esquisatto M.A.M., Rodrigues R.A.F., Gomes L., Pimentel E.R. (2015). Effect of *Calendula officinalis* cream on achilles tendon healing. Anat. Rec..

[52] Akhtar N., Khan B.A., Haji M., Khan S., Ahmad M., Rasool F., Mahmood T., Rasul A. (2011). Evaluation of various functional skin parameters using a topical cream of calendula officinalis extract. Afr. J. Pharm. Pharmacol..

[53] Bernatoniene J., Masteikova R., Davalgiene J., Peciura R., Gauryliene R., Bernatoniene R., Majiene D., Lazauskas R., Civinskiene G., Velziene S. (2011). Topical application of *Calendula officinalis* (L.): Formulation and evaluation of hydrophilic cream with antioxidant activity. J. Med. Plants Res..

[54] Varka E.-M., Tsatsaroni E., Xristoforidou N., Darda A.-M. (2012). Stability Study of O/W Cosmetic Emulsions Using *Rosmarinus officinalis* and *Calendula officinalis* Extracts. Open J. Appl. Sci..

[55] Singleton V.L., Rossi J.A.J. (1965). Colorimetry to total phenolics with phosphomolybdic acid reagents. Am. J. Enol. Vitic..

[56] Boun H.R., Huxsoll C.C. (1991). Control of Minimally Processed Carrot (*Daucus carota*) Surface Discoloration Caused by Abrasion Peeling. J. Food Sci..

[57] Han J.H., Floros J.D. (1997). Casting antimicrobial packaging films and measuring their physical properties and antimicrobial activity. J. Plast. Film Sheeting.

[58] Aguirre-Loredo R.Y., Rodríguez-Hernández A.I., Morales-Sánchez E., Gómez-Aldapa C.A., Velazquez G. (2016). Effect of equilibrium moisture content on barrier, mechanical and thermal properties of chitosan films. Food Chem..

[59] Kozlowska J., Tylkowski B., Stachowiak N., Prus-Walendziak W. (2020). Controlling the skin barrier quality through the application of polymeric films containing microspheres with encapsulated plant extract. Processes.

[60] Stachowiak N., Kowalonek J., Kozlowska J. (2020). Effect of plasticizer and surfactant on the properties of poly (vinyl alcohol)/chitosan films. Int. J. Biol. Macromol..

[61] Aydogdu A., Radke C.J., Bezci S., Kirtil E. (2020). Characterization of curcumin incorporated guar gum/orange oil antimicrobial emulsion films. Int. J. Biol. Macromol..

[62] Shaw N.B., Monahan F.J., O’Riordan E.D., O’Sullivan M. (2002). Physical properties of WPI films plasticized with glycerol, xylitol, or sorbitol. J. Food Sci..

[63] Zhang P., Zhao Y., Shi Q. (2016). Characterization of a novel edible film based on gum ghatti: Effect of plasticizer type and concentration. Carbohydr. Polym..

[64] Sanyang M.L., Sapuan S.M., Jawaid M., Ishak M.R., Sahari J. (2016). Effect of plasticizer type and concentration on physical properties of biodegradable films based on sugar palm (*Arenga pinnata*) starch for food packaging. J. Food Sci. Technol..

[65] Fabra M.J., Talens P., Chiralt A. (2008). Tensile properties and water vapor permeability of sodium caseinate films containing oleic acid-beeswax mixtures. J. Food Eng..

[66] Han J.H., Seo G.H., Park I.M., Kim G.N., Lee D.S. (2006). Physical and mechanical properties of pea starch edible films containing beeswax emulsions. J. Food Sci..

[67] Limpisophon K., Tanaka M., Osako K. (2010). Characterisation of gelatin-fatty acid emulsion films based on blue shark (*Prionace glauca*) skin gelatin. Food Chem..

[68] Heinrich U., Koop U., Leneveu-Duchemin M.C., Osterrieder K., Bielfeldt S., Chkarnat C., Degwert J., Häntschel D., Jaspers S., Nissen H.P. (2003). Multicentre comparison of skin hydration in terms of physical-, physiological- and product-dependent parameters by the capacitive method (Corneometer CM 825). Int. J. Cosmet. Sci..

[69] Velasco M.V.R., Vieira R.P., Fernandes A.R., Dario M.F., Pinto C.A.S.O., Pedriali C.A., Kaneko T.M., Baby A.R. (2014). Short-term clinical of peel-off facial mask moisturizers. Int. J. Cosmet. Sci..

